# Editorial: Frailty and Sarcopenia in Various Cachectic Kidney Diseases

**DOI:** 10.3389/fmed.2020.627485

**Published:** 2021-01-12

**Authors:** Yoshiyuki Morishita, Kunihiro Sakuma, Chia-Ter Chao

**Affiliations:** ^1^Division of Nephrology, Department of Integrated Medicine, Saitama Medical Center, Jichi Medical University, Saitama, Japan; ^2^Institute for Liberal Arts, Environment and Society, Tokyo Institute of Technology, Tokyo, Japan; ^3^Division of Nephrology, Department of Internal Medicine, National Taiwan University Hospital BeiHu Branch, Taipei, Taiwan; ^4^Division of Nephrology, Department of Internal Medicine, National Taiwan University College of Medicine, Taipei, Taiwan; ^5^Graduate Institute of Toxicology, National Taiwan University College of Medicine, Taipei, Taiwan

**Keywords:** chronic kidney disease, cachexia, frailty, sarcopenia, biomarker

Cachexia is a complex metabolic disorder and leads to many problems, including muscle wasting and extreme weight loss. Patients who have a cachectic condition have a high probability of developing frailty and sarcopenia. These patients also have a high probability of developing geriatric syndromes, which were first described in the aged population, but were later found to be prevalent among patients who have various diseases. Patients with chronic kidney disease (CKD) have an increased risk of cachexia, and may develop frailty and sarcopenia. Frailty and sarcopenia contribute to adverse outcomes in patients with CKD, regardless of their baseline renal function (1). Additionally, various diseases that are complicated by CKD, such as cancer, chronic heart failure, and chronic obstructive pulmonary disease, may contribute to accelerating development for frailty and sarcopenia in this population.

To improve these problems regarding frailty and sarcopenia in CKD, evidence on the mechanism, biomarkers, and treatments of frailty and sarcopenia in CKD is considered to be urgently required. Therefore, we focus on studies that contribute to promoting a more in-depth understanding of mechanisms, risk factors, and clinical implications of frailty and sarcopenia in CKD in this special issue.

Chang et al. examined the combined effect of the malnutrition–inflammation cachexia score, which is an inflammation marker, and the degree of autonomic stability on the survival of patients with end-stage renal disease. These authors showed that both factors elevated the risk of mortality in this population. Their findings support the clinical importance of assessing the severity of inflammation in this population. Ito et al. found that skeletal muscle mass loss was an independent risk factor for a decrease in bone mineral density in patients undergoing hemodialysis. This study indicates that preservation of skeletal muscle mass may be important for preventing a decrease in bone mineral density in this population. Kaneko et al. showed that the prevalence of serum zinc deficiency was high and serum zinc concentrations were correlated with serum ferritin concentrations in patients undergoing peritoneal dialysis. Zinc deficiency induces various pathological conditions, such as dermatitis, anemia, susceptibility to infection, and gastrointestinal disorders. However, serum zinc concentrations are only measured when patients show characteristic symptoms. Clinicians may note potential zinc deficiency in patients with iron deficiency among those undergoing peritoneal dialysis.

Two studies showed interesting findings associated with frailty and sarcopenia. Li et al. examined the red cell distribution width, which showed an independent association with frailty in community-dwelling older adults. Yanai et al. small non-coding RNAs (microRNAs) that inhibit translation of target mRNA in sarcopenia. Since the findings of these two studies did not limit in CKD, further studies in these contents in CKD may be interesting.

In conclusion, interdisciplinary collaboration studies, incorporating not only bench models and clinical medicine but also geriatrics and nephrology, as in this special issue, are likely to have a major effect on the care of frailty and sarcopenia in CKD.

## Author Contributions

YM compiled the contributions from all authors/editors. All authors/editors approved the final version of the manuscript.

## Conflict of Interest

The authors declare that the research was conducted in the absence of any commercial or financial relationships that could be construed as a potential conflict of interest.
